# Hypertension-associated mitochondrial DNA 4401A>G mutation caused the aberrant processing of tRNA^Met^, all 8 tRNAs and ND6 mRNA in the light-strand transcript

**DOI:** 10.1093/nar/gkz742

**Published:** 2019-08-28

**Authors:** Xiaoxu Zhao, Limei Cui, Yun Xiao, Qin Mao, Maerhaba Aishanjiang, Wanzhong Kong, Yuqi Liu, Hong Chen, Fang Hong, Zidong Jia, Meng Wang, Pingping Jiang, Min-Xin Guan

**Affiliations:** 1 Division of Medical Genetics and Genomics, The Children's Hospital, Zhejiang University School of Medicine, Hangzhou, Zhejiang 310058, China; 2 Institute of Genetics, and Department of Human Genetics, Zhejiang University School of Medicine, Hangzhou, Zhejiang 310058, China; 3 Department of Clinical Laboratory, Wenzhou Traditional Chinese Medicine Hospital, Wenzhou, Zhejiang 325000, China; 4 Cardiac Department, Chinese PLA General Hospital, Beijing 100853, China; 5 Emergy Medicine Department, Ningbo First Hospital, Zhejiang University School of Medicine, Ningbo, Zhejiang 315000, China; 6 Key lab of Reproductive Genetics, Ministry of Education of PRC, Zhejiang University, Hangzhou, Zhejiang 310058, China; 7 Joint Institute of Genetics and Genome Medicine between Zhejiang University and University of Toronto, Hangzhou, Zhejiang 310058, China

## Abstract

Mitochondrial tRNA processing defects were associated with human diseases but their pathophysiology remains elusively. The hypertension-associated m.4401A>G mutation resided at a spacer between mitochondrial tRNA^Met^ and tRNA^Gln^ genes. An *in vitro* processing experiment revealed that the m.4401A>G mutation caused 59% and 69% decreases in the 5′ end processing efficiency of tRNA^Gln^ and tRNA^Met^ precursors, catalyzed by RNase P, respectively. Using human umbilical vein endothelial cells-derived cybrids, we demonstrated that the m.4401A>G mutation caused the decreases of all 8 tRNAs and ND6 and increases of longer and uncleaved precursors from the Light-strand transcript. Conversely, the m.4401A>G mutation yielded the reduced levels of tRNA^Met^ level but did not change the levels of other 13 tRNAs, 12 mRNAs including ND1, 12S rRNA and 16S rRNA from the Heavy-strand transcript. These implicated the asymmetrical processing mechanisms of H-strand and L-strand polycistronic transcripts. The tRNA processing defects play the determined roles in the impairing mitochondrial translation, respiratory deficiency, diminishing membrane potential, increasing production of reactive oxygen species and altering autophagy. Furthermore, the m.4401A>G mutation altered the angiogenesis, evidenced by aberrant wound regeneration and weaken tube formation in mutant cybrids. Our findings provide new insights into the pathophysiology of hypertension arising from mitochondrial tRNA processing defects.

## INTRODUCTION

**Figure 1.**
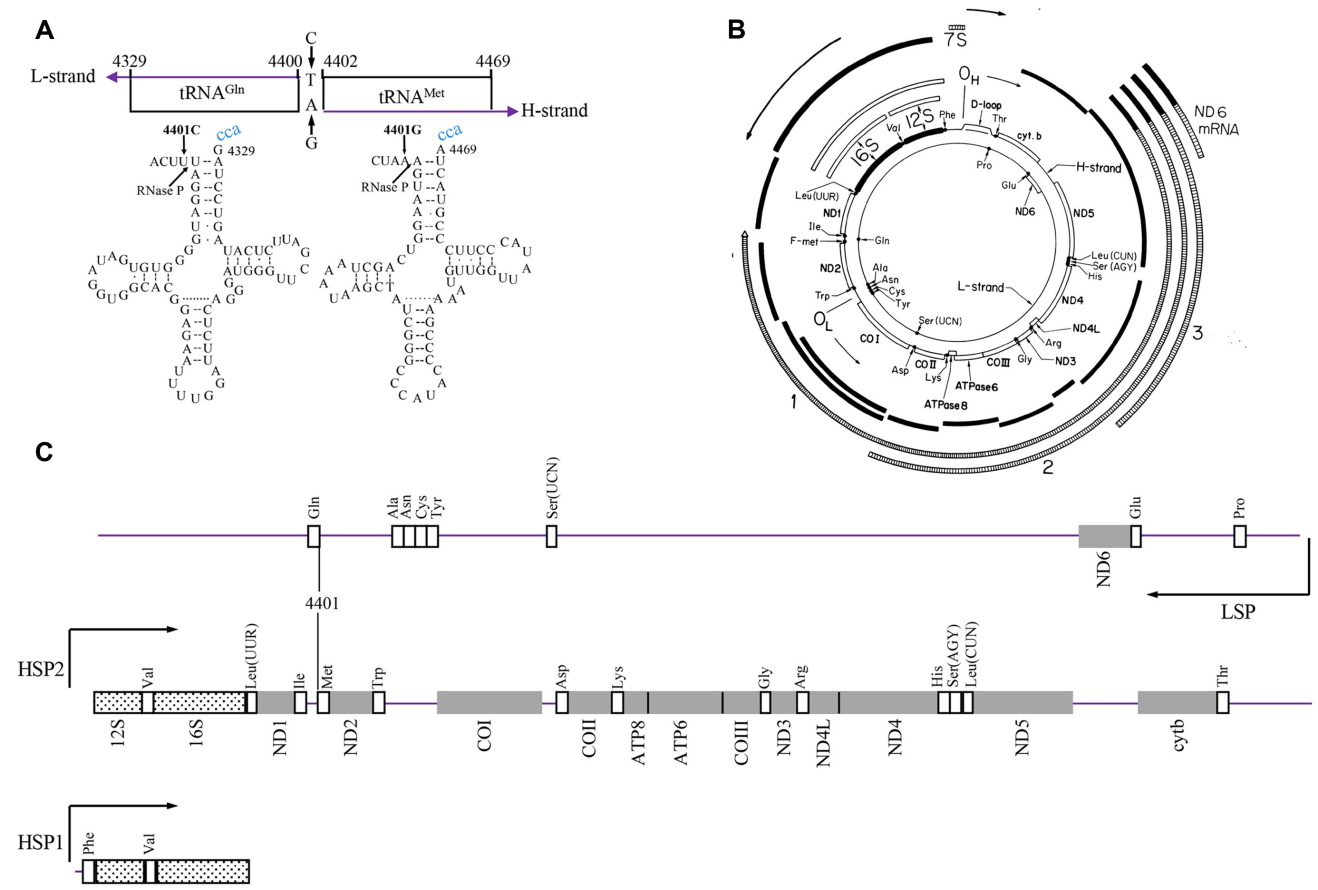
A schema of location of m.4401A>G mutation in the precursors of tRNA^Met^ and tRNA^Gln^, genetic and transcription map of human mitochondria. (**A**) Cloverleaf structures of mitochondrial tRNA^Met^ and tRNA^Gln^ are derived from Florentz *et al.* (31). Processing sites in the tRNA^Met^ and tRNA^Gln^ precursors were determined for RNase P. Arrow indicates the position of the m.4401A>G mutation. (**B**) Genetic and transcription maps of mitochondrial genomes were derived from Guan *et al.* (5). The two inner circles show the positions of 12S and 16S rRNA (black bars), of the 13 reading frames (ND1, ND2, ND3, ND4, ND4L, ND5, and ND6, COI, COII, COIII, cytb, A6 and A8) (white bars) and of 22 tRNA (solid circles). (**C**) Three polycistronic RNA transcripts (7,8). The transcription of L-strand promoter (LSP) resulted in a near genomic length primary transcript encoding eight tRNAs including tRNA^Gln^, tRNA^Ser(UCN)^ and ND6. The transcription of H-strand promoter 1 (HSP1) generated the short transcript containing tRNA^Phe^, tRNA^Val^, 12S rRNA and 16S rRNA, while the transcription from HSP2 produced an almost genome transcript consisting of 12S rRNA, 16S rRNA, 12 mRNAs and 14 tRNAs including tRNA^Met^, tRNA^Lys^ and tRNA^Gly^. RNA sequences are represented as follows: rRNAs as hashed boxes, mRNAs as gray boxes and tRNAs as white boxes.

Defects in mitochondrial RNA processing have been associated with human diseases including neurological disorders, deafness, hypertrophic cardiomyopathy and hypertension (1–6). Human mitochondrial 22 tRNAs, together with 13 mRNA coding 13 polypeptides for essential subunits of oxidative phosphorylation system (OXPHOS) and 2 rRNAs, were transcribed as the polycistronic heavy (H) and light (L) strand transcripts, from the mitochondrial genome (mtDNA) (7–11). As shown in Figure 1, the transcription of L-strand promoter (LSP) resulted in a near genomic length primary transcript encoding eight tRNAs including tRNA^Gln^, tRNA^Ser(UCN)^ and ND6 (11,12). Classically, the transcription of H-strand promoter 1 (HSP1) generated the short transcript containing tRNA^Phe^, tRNA^Val^, 12S rRNA and 16S rRNA, while the transcription from HSP2 produced an almost genome transcript consisting of 12S rRNA, 16S rRNA, 12 mRNAs and 14 tRNAs including tRNA^Met^, tRNA^Lys^ and tRNA^Gly^ (8,11,12). However, the existence of HSP2 as functional promoter *in vivo* is still questionable (13). The processing of mitochondrial tRNAs from the primary transcripts required the precise cleavage of tRNAs at their 5′ ends catalyzed by RNase P, which consists of three subunits, encoded by *MRPP1, MRPP2* and *MARPP3*, and 3′ terminal mediated by RNase Z, encoded by *ELAC2* (14–17). This processing resulted in the release of the individual translation-competents: mRNAs, tRNAs and rRNAs from their polycistronic precursors. The aberrant 5′ end tRNA processing caused by mutations in the *MRRP1* or *MRRP2* resulted in mitochondrial dysfunctions leading to clinical phenotypes (18–20), while the defects in the 3′ end tRNA processing caused by mutations in ELAC2 were responsible for cardiomyopathy (21,22). The 5′ and 3′ end processing defects arising from mitochondrial tRNA mutations also caused human diseases. The deafness-associated m.7445T>C mutation in the precursor of tRNA^Ser(UCN)^ and cardiomyopathies-associated tRNA^Ile^ 4269A>G and 4295A>G mutations and tRNA^His^ 12192G>A mutation perturbed the 3′ end processing of corresponding tRNA precursors (5,23–25). Furthermore, the mitochondrial encephalomyopathy, lactic acidosis, stroke-like symptoms (MELAS)-associated 3243A>G mutation and mitochondrial myopathy-associated 3302A>G mutation in the tRNA^Leu(UUR)^ caused the 5′ end aberrant processing of tRNA^Leu(UUR)^ and accumulation of RNA precursors (26,27). Recently, we identified the hypertension-associated tRNA^Ile^ 4263A>G, tRNA^Ala^ 5655A>G, tRNA^Trp^ 5512A>G mutations at the 5′ end (conventional position 1) of corresponding tRNAs, and m.4401A>G mutation at the junction of tRNA^Met^ and tRNA^Gln^ genes (6,28–30). An *in vitro* processing analysis demonstrated that the m.4263A>G and m.5655A>G mutations reduced the 5′ end processing efficiencies of tRNA^Ile^ and tRNA^Ala^ precursors, catalyzed by RNase P, respectively (28,29). However, the pathophysiology underlying these tRNA mutations, specifically the tissue specific effects, remains elusively.

As shown in Figure 1, the m.4401A>G mutation occurred in the four genetically unrelated Chinese hypertensive pedigrees was localized at the spacer immediately to the 5′ ends of tRNA^Met^ at the H-strand transcript and tRNA^Gln^ at the L-strand transcript (6,30,31). We therefore hypothesized that the m.4401A>G mutation altered the 5′ end processing of tRNA^Met^ and the tRNA^Gln^ precursors, catalyzed by RNase P. It was also anticipated that the aberrant tRNA metabolism led to the impairment of mitochondrial translation, respiration deficiency, oxidative stress, uncoupling of the oxidative pathways for ATP synthesis, and subsequent failure of cellular energetic processes. In the previous study, the lymphoblastoid cell lines harboring the m.4401A>G mutation displayed the reductions in the steady-state levels of tRNA^Met^ and tRNA^Gln^, impairment of mitochondrial translation and respiratory deficiency (30). However, the tissue specific effects of m.4401A>G mutation on the development of hypertension remains elusively. The pathogenic mechanism behind the tissue-specific manifestations is likely to involve the cell type-specific mitochondrial functions. The lack of animal model for pathogenic mtDNA mutations has hampered mechanistic studies. Human umbilical vein endothelial cells (HUVECs) are a valuable *in vitro* model for the study of endothelial cell physiology and pathology at the cardiovascular level (32,33). In this study, we generated the HUVECs derived cybrids by transferring mitochondria from lymphoblastoid cell lines derived from a Chinese family carrying the m.4401A>G mutation and from a control subject lacking the mutation into mtDNA-less HUVECs (33–36). These cybrid lines were analyzed for the effects of the m.4401A>G mutation on the processing of tRNA and mRNA, the stability of tRNA, mitochondrial translation, respiration, mitochondrial membrane potential, production of reactive oxidative species (ROS) and autophagy. Furthermore, these cybrid lines were further characterized by examining wound healing and tube formation to evaluate the impact of m.4401A>G mutation-induced alterations on angiogenic properties.

## MATERIALS AND METHODS

### Cell lines and culture conditions

Immortalized lymphoblastoid cell lines were generated from affected matrilineal relatives (II-1, III-3) of a Chinese family carrying the homoplasmic m.4401A>G mutation (30) and one genetically unrelated Chinese control individual (C19) belonging to the similar mtDNA haplogroup C4 but lacking the mutation. The sequences of whole mitochondrial genomes in these cell lines were determined as detailed elsewhere (37) (Supplementary Table S1). Immortalized lymphoblastoid cell lines were grown in RPMI 1640 medium with 10% FBS. 143B.TK^−^ cell line was grown in DMEM (Life Technologies) (containing 4.5 mg of glucose and 0.11 mg of pyruvate/ml), supplemented with 100 μg of BrdU/ml and 5% FBS. The mtDNA-less ρ°206 cell line, derived from 143B.TK^−^ (36) was grown under the same conditions as the parental line, except for the addition of 50 μg of uridine/ml. HUVECs were grown in endothelial basal medium (ScienCell) supplemented with endothelial cell growth supplement (ECGS) and 5% fetal bovine serum (FBS) (33).

The HUVECs-less-mtDNA lines were produced as detailed elsewhere (33,34). Transformation by cytoplasts of HUVECs-less-mtDNA lines using enucleated lymphoblastoid cells from one affected subject (III-3) and one control individual (C19) was performed as described elsewhere (33–36). Resultant cybrid clones were examined for the presence and degree of m.4401A>G mutation and the HUVECs specific m.4336T>C variant as detailed previously (Supplemental Figure S1) (30,33). Cybrids lacking m.4336T>C variant and harboring the homoplasmic m.4401A>G mutation in mutant clones and lacking both m.4336T>C and m.4401A>G mutations in control clones were determined for the copy numbers of mtDNA, as described elsewhere (Supplemental Figure S2) (36). Three cybrid cell clones derived from each donor cell line with similar mtDNA copy numbers were used for the biochemical characterization described below. All cybrid cell lines constructed with enucleated lymphoblastoid cell lines were maintained in the same medium as the parental HUVECs.

### Sequencing of 5′- and 3′-end proximal segments of tRNA^Met^ and tRNA^Gln^

The 5′ and 3′ ends of tRNA^Met^ and tRNA^Gln^ from the control cell line C19 and mutant cell line III-3 were sequenced after cDNA synthesis, PCR amplification, and cloning, as detailed elsewhere (38). First, total mitochondrial tRNA was circularized by incubation in the presence of T4 RNA ligase (Promega) to ligate the 3′ and 5′ ends of tRNAs. Then, complementary DNA chains of tRNA^Met^ and tRNA^Gln^ were synthesized using reverse transcriptase after annealing the circular tRNA to the specific oligodeoxynucleotides MET1 (5′-TATGGGCCCGATAGCTTATTTAGCT-3′) and GLN1 (5′CAAAATTCTCCGTGCCACCTATCA-3′), respectively. The second strands of these cDNAs were then synthesized by using primers MET2 (5′-CCCCGAAAATGTTGGTTATACCCTT-3′), GLN2 (5′-GATTCTCAGGGATGGGTTCGATT-3′), respectively. The artificial tDNAs were then amplified by PCR, using above primers, respectively. Those resultant PCR products were cloned in the TA vector (Invitrogen), and eight clones of each control cell tDNA and III-3-1′s tDNA were analyzed by Sanger sequence.

### Mitochondrial RNase P assay

The wild type and mutant precursors of tRNA^Met^ corresponding to mtDNA at positions 4365 (5′) to 4469 (3′), and tRNA^Gln^ at mtDNA positions 4438 (5′) to 4329 (3′) were cloned into the pCRII-TOPO vector carrying SP6 and T7 promoters (Clontech). After *Hind*III digestion, the labeled RNA substrates (104 nt for tRNA^Met^ and 109 nt for tRNA^Gln^) were transcribed with T7 RNA polymerase, in the presence of 10 μM ATP, CTP, GTP and UTP, pH 7.5 and 10 units RNase inhibitor at 20°C. Transcripts were purified by denaturing polyacrylamide gel electrophoresis (PAGE) (7 M urea, 8% polyacrylamide/bisacrylamide [19:1]) and were dissolved in 1 mM EDTA. Mitochondrial RNase P was reconstituted from purified recombinant proteins MRPP1, MRPP2 and MRPP3 as described previously (15,28). Processing assays were carried out in parallel for wild type and mutant substrates in 12 μl reaction mixtures containing 30 mM TrisCl, 30 mM NaCl, 4.5 mM MgCl_2_, 200 μg/ml BSA, RNA substrates and 25 nM RNase P, at 30°C. After 1, 5, 10, 20, 50 and 60 min, aliquots were withdrawn and stopped by addition of five loading buffer (85% formamide, 10 mM EDTA). Reaction products were resolved via denaturing PAGE, then electroblotted onto a nylon membrane (Roche) and hybridized with digoxigenin (DIG)-labeled oligodeoxynucleotide probes for tRNA^Met^ precursor (5′-TAGGATGGGGTGTGATAGGTGGCACGGAGAATTTT-3′) and tRNA^Gln^ precursor (5′-AGTAAGGTCAGCTAAATAAGCTATCGGGCCCATACCC-3′). DIG-labeled probes were generated by using DIG-oligonucleotide Tailing kit (Roche). The hybridization and quantification of density in each band were performed as detailed previously (28).

### Mitochondrial RNA analysis

Total cellular and mitochondrial RNAs were obtained by using TOTALLY RNA™ kit (Ambion) from intact cells or mitochondria isolated from the various cell lines (∼4 × 10^7^ cells), as detailed elsewhere (39). For tRNA Northern blot analysis, 2 μg of total mitochondrial RNAs were electrophoresed through a 10% polyacrylamide/8 M urea gel in Tris–borate–EDTA buffer (TBE) after heating the samples at 65°C for 10 min, and then electroblotted onto a positively charged nylon membrane (Roche) for the hybridization analysis with DIG-labeled oligodeoxynucleotide probes, respectively. Oligodeoxynucleosides used for a set of DIG-labeled probes of 22 mitochondrial tRNAs and 5S rRNA were listed in the Supplemental Table S2. The hybridization and quantification of density in each band were performed as detailed previously (40–44).

For mRNA Northern blot analysis, 8 μg of total cellular RNAs were fractionated by electrophoresis through a 1.5% agarose-formaldehyde gel, transferred onto a positively charged membrane (Roche), and hybridized with a set of DIG-labeled RNA probes: ND6, ND1, COX1, COX2, CYTB, 12S rRNA, 16S rRNA, and β-actin as a control, respectively. Probes were synthesized on the corresponding restriction enzyme linearized plasmid using a DIG RNA Labeling kit (Roche). The plasmids used for RNA probes were constructed by PCR-amplifying fragments of ND6 (positions 14343 to 14618), ND1 (positions 3506–3839), COX1 (positions 7146–7425), COX2 (positions 7823–8156), CYTB (positions 14824–15208), 12S rRNA (positions 1201–1235), 16S rRNA (positions 2245–2635), and β-actin (positions 69–618, NM_001101.5) and cloning these fragments into the pCRII-TOPO vector (45).

### Western blot analysis

Western blotting analysis was carried out as detailed elsewhere (43,44). Twenty micrograms of total cellular proteins obtained from various cell lines were denatured and loaded on sodium dodecyl sulfate (SDS) polyacrylamide gels. Afterward, the gels were electroblotted onto polyvinylidene difluoride (PVDF) membrane for hybridization. The antibodies used for this investigation were from Abcam [ND1(ab74257), ND5 (ab92624), ND6 (ab81212), CO2 (ab110258), Tom20 (ab56783), p62 (ab56416) and Total OXPHOS Human WB Antibody Cocktail (ab110411)], Proteintech [(CYTB (55090-1-AP), ND2 (19704-1-AP), ATP8 (26723-1-AP), and β-actin (20536-1-AP)], Novus [ND4 (NBP2-47365)], Cell Signaling Technology [LC3A/B (CST,#4108)]. Peroxidase Affini Pure goat anti-mouse IgG and goat anti-rabbit IgG (Jackson) were used as secondary antibodies, and protein signals were detected using the ECL system (CWBIO). Antibodies against above human mtDNA encoding proteins were validated using 143B.TK^−^ cell line and mtDNA-less ρ°206 cell line (Supplemental Figure S3). Quantification of density in each band was performed as described previously (43,44).

### Measurements of oxygen consumption

The rates of oxygen consumption (OCR) in various cell lines were determined with a Seahorse Bioscience XF-96 extracellular flux analyzer (Seahorse Bioscience), as detailed elsewhere (46). Cells were seeded at a density of 1 × 10^4^ cells per well on Seahorse XF96 polystyrene tissue culture plates (Seahorse Bioscience). Inhibitors were used at the following concentrations: Oligomycin (to inhibit the ATP synthase) (1.5 μM), Carbonyl cyanide 4-trifluoromethoxy-phenylhydrazone (FCCP) (to uncouple the mitochondrial inner membrane and allow for maximum electron flux through the electron transfer chain) (0.15μM), Antimycin A (to inhibit complex III) (5 μM) and Rotenone (to inhibit complex I) (1 μM).

### Assessment of mitochondrial membrane potential

Mitochondrial membrane potential from various cell lines was examined with JC-10 Assay Kit-Flow Cytometry (Abcam) following general manufacturer's recommendations with some modifications, as described previously (33,47).

### Analysis of mitochondrial ROS production

The levels of mitochondrial reactive oxygen species (ROS) generation in various cell lines were measured using MitoSOX assay as detailed elsewhere (29,33,48).

### Analysis of autophagy

The fluorescence-based cytometry to analyze the level of mitophagy was performed using CYTO-ID® Autophagy Detection Kit (Enzo), as detailed elsewhere (49,50). In brief, ∼3 × 10^5^ cells of each cybrid cell line were incubated with the endothelial basal medium (ScienCell) in the absence and presence of 500 nM rapamycin (inducers of autophagy) and 10 μM chloroquine (lysosomal inhibitor) at 37°C for 18 h, and spin down and washed with PBS. The resultant samples were resuspended with CYTO-ID^®^-Green reagent and analyzed using a Novocyte flow cytometer (ACEA Biosciences) in FITC channel.

### Wound healing assay

Wound healing assays were performed as detailed elsewhere (33,51).

### Tube formation assay

A tube formation assay using growth factor-reduced Matrigel was carried out as detailed previously (33,52,53).

### Statistical analysis

Statistical analysis was performed by the unpaired, two-tailed Student's *t*-test contained in Microsoft Office Excel (version 2013) and GraphPad Prism (v5.04, www.graphpad.com). *P* indicates the significance, according to the t-test, of the difference between mutant and control mean. Differences were considered significant at a *P* < 0.05.

## RESULTS

### No effect of m.4401A>G mutation on the coding sequences of tRNA^Met^ and tRNA^Gln^

To examine if the m.4401A>G mutation affects the coding sequences of tRNA^Met^ and tRNA^Gln^, the 5′ and 3′ ends of the mitochondrial tRNA^Met^ and tRNA^Gln^ from the control cell line C19 and mutant cell line III-3 were analyzed by Sanger sequence after cDNA synthesis, PCR amplification, and cloning, as described elsewhere (28,38). Indeed, both sequences of tRNA^Met^ and tRNA^Gln^ from mutant and control cell lines lacked nt 4401. Furthermore, no differences was observed between wild type and mutant sequences of tRNA^Met^ and tRNA^Gln^. These result indicated that the m.4401A>G mutation did not affect the coding sequences of tRNA^Met^ and tRNA^Gln^.

### Impaired the 5′ end processing of tRNA^Met^ and tRNA^Gln^ precursors

**Figure 2.**
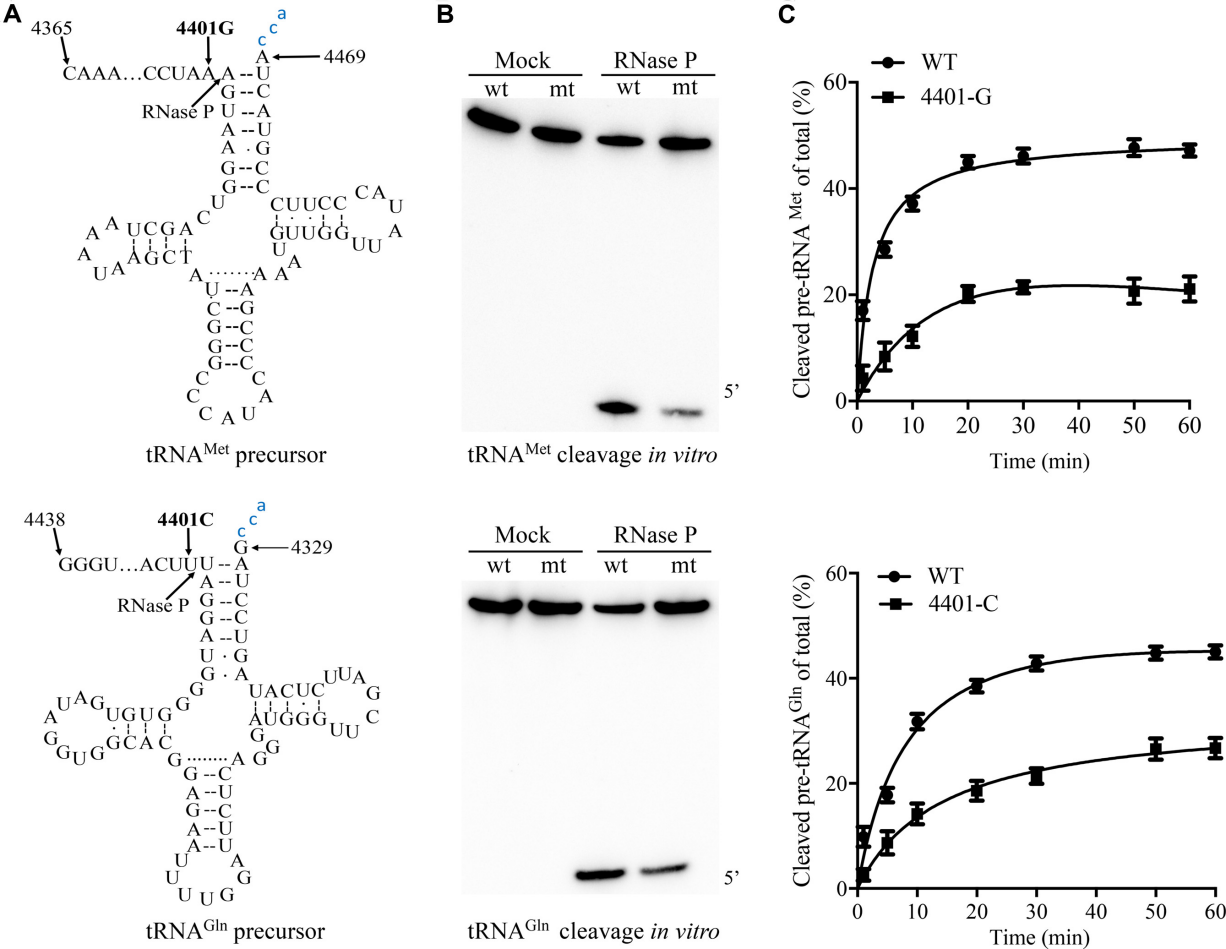
*In vitro* assay for the processing of mitochondrial tRNA^Met^ and tRNA^Gln^ precursors. (**A**) Mitochondrial tRNA^Met^ and tRNA^Gln^ precursors. Thirty-seven and 38 nucleotides (nt) of 5′ end leaders of tRNA^Met^ and tRNA^Gln^ were shown, including the m.4401A>G substitution. (**B**) *In vitro* processing assays. Processing assays with mitochondrial RNase P were carried out in parallel for wild type and mutant substrates. Samples were withdrawn and stopped after 1, 5, 10, 20, 50 or 60 min, respectively. Reaction products were resolved by denaturing polyacrylamide gel electrophoresis and reacted with a chemiluminescent substrate CDP-Star™ to detect the chemiluminescent. The graph shows the results of a representative experiment at 10 min reaction. (**C**) Relative processing efficiencies of tRNA^Met^ and tRNA^Gln^ precursors catalyzed by RNase P. The relative processing efficiencies were calculated from the initial phase of the reaction. The calculations were based on five independent determinations. The error bars indicate two standard errors of the mean (SEM).

We employed an *in vitro* processing system to investigate if the primary defect arising from the m.4401A>G mutation is the aberrant 5′ end processing of tRNA^Met^ at the H-strand transcripts and tRNA^Gln^ at the L-strand-transcripts, catalyzed by RNase P. For this purpose, the wild-type and mutant tRNA^Met^ and tRNA^Gln^ precursors corresponding to mtDNA at positions 4365 to 4469 and 4438 to 4329 (Figure 2A) were prepared by *in vitro* transcription, respectively. To analyze the *in vitro* processing kinetics, the wild type and mutant tRNA^Met^ and tRNA^Gln^ precursors were incubated with mitochondrial RNase P, which was reconstituted from purified recombinant proteins MRPP1, MRPP2 and MRPP3, at various time courses (15,28,29). No qualitative processing alterations of the mutant tRNA^Met^ and tRNA^Gln^ precursors were observed, but the processing efficiencies of the mutant tRNA^Met^ and tRNA^Gln^ transcripts were markedly reduced, as compared with those of wild type counterparts (Figure 2B). The processing efficiencies of mutant tRNA^Met^ and tRNA^Gln^ transcripts catalyzed by RNase P were ∼31% and ∼41% of those in their wild type counterparts (Figure 2C), respectively. These results demonstrated that the m.4401A>G mutation altered the 5′ end processing of tRNA^Met^ and tRNA^Gln^ precursors.

### Reduced levels of tRNA ^Met^ from H-strand transcript and all 8 tRNAs from L-strand transcript

**Figure 3.**
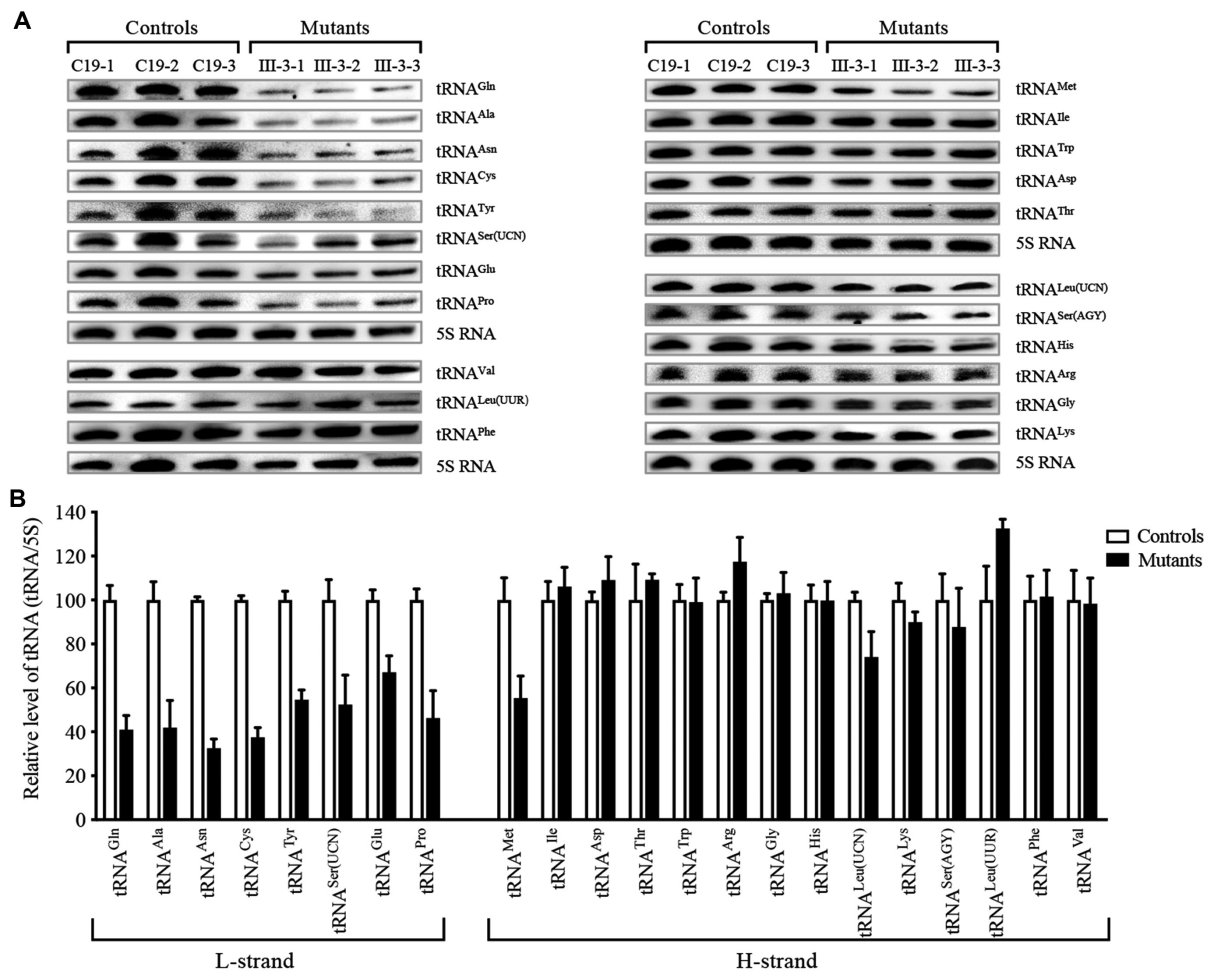
Northern blot analysis of mitochondrial tRNAs. (**A**) Two microgram of total mitochondrial RNAs from the various cell lines were electrophoresed through a denaturing polyacrylamide gel, were electroblotted, and were hybridized with DIG-labeled oligonucleotide probes specific for tRNA^Gln^, tRNA^Ala^, tRNA^Asn^, tRNA^Cys^, tRNA^Tyr^, tRNA^Ser(UCN)^, tRNA^Glu^, and tRNA^Pro^ from the L-strand transcripts, tRNA^Met^, tRNA^Thr^, tRNA^Trp^, tRNA^Ile^, tRNA^Leu(UUR)^, tRNA^Leu(CUN)^, tRNA^Lys^, tRNA^Ser(AGY)^, tRNA^His^, tRNA^Gly^, tRNA^Phe^, tRNA^Asp^, tRNA^Asn^ and tRNA^Val^ from the H-strand transcripts, and 5S rRNA, respectively. (**B**) Quantification of the tRNA levels. Average relative each tRNA content per cell was normalized to the average content per cell of 5S rRNA in the control and mutant cell lines, respectively. The values for the latter are expressed as percentages of the average values for the control cell lines. The calculations were based on three independent determinations in each cell line. The error bars indicate two standard errors of the mean (SEM). *P* indicates the significance, according to the t-test, of the differences between mutant and control cell lines.

Among 22 mitochondrial tRNAs, 8 tRNAs such as tRNA^Glu^ and tRNA^A1a^ genes resided on the L-strand transcript, the remaining tRNA genes including, tRNA^Met^, tRNA^Leu(UUR)^, tRNA^Asp^ and tRNA^Gly^ are located at the H-strand transcript (7–9). It was hypothesized that the altered 5′ end processing of tRNA^Met^ and tRNA^Gln^ by the m.4401A>G mutation altered the mitochondrial tRNA metabolisms. For this purpose, we subjected total mitochondrial RNAs from mutant and control cell lines to Northern blots and hybridized them with DIG-labeled oligodeoxynucleotide probes for 14 tRNAs including tRNA^Met^, tRNA^Lys^, tRNA^Leu(UUR)^, tRNA^Ser(AGY)^ derived from the H-strand transcripts and 8 tRNAs including tRNA^Gln^, derived from the L-strand transcripts (Supplemental Table S2), respectively. As shown in Figure 3, the average levels of tRNA^Gln^ and tRNA^Met^ in three mutant cybrids were 41% and 55% (*P* < 0.01) of the mean values of three control cybrids, respectively. Strikingly, the average steady-state levels of other 7 tRNAs from the L-strand transcript were significantly decreased. Especially, the average levels of tRNA^Ala^, tRNA^Asn^, tRNA^Cys^, tRNA^Tyr^, tRNA^Ser(UCN)^, tRNA^Glu^ and tRNA^Pro^ in three mutant cybrids were 42%, 33%, 38%, 55%, 52%, 67% and 46% of those in three control cybrids, respectively. Interestingly, the average levels of 8 tRNAs from L-strand transcript in the mutant cell lines were 46.75% of those in the control cell lines. In contrast, the steady-state levels of other 13 tRNAs, such as tRNA^Lys^, tRNA^Leu(UUR)^ and tRNA^Ser(AGY)^ from H-strand transcript in the mutant cybrids were comparable with those in three control cybrids. Furthermore, these results were verified by tRNA Northern blot analysis using mutant and control immortalized lymphoblastoid cell lines (Supplemental Figure S4). These data indicated that the m.4401A>G mutation led to the metabolic defects of all 8 tRNAs, derived from L-strand transcript and tRNA^Met^ from H-strand transcript.

### Aberrant processing of ND6 mRNA precursors

**Figure 4.**
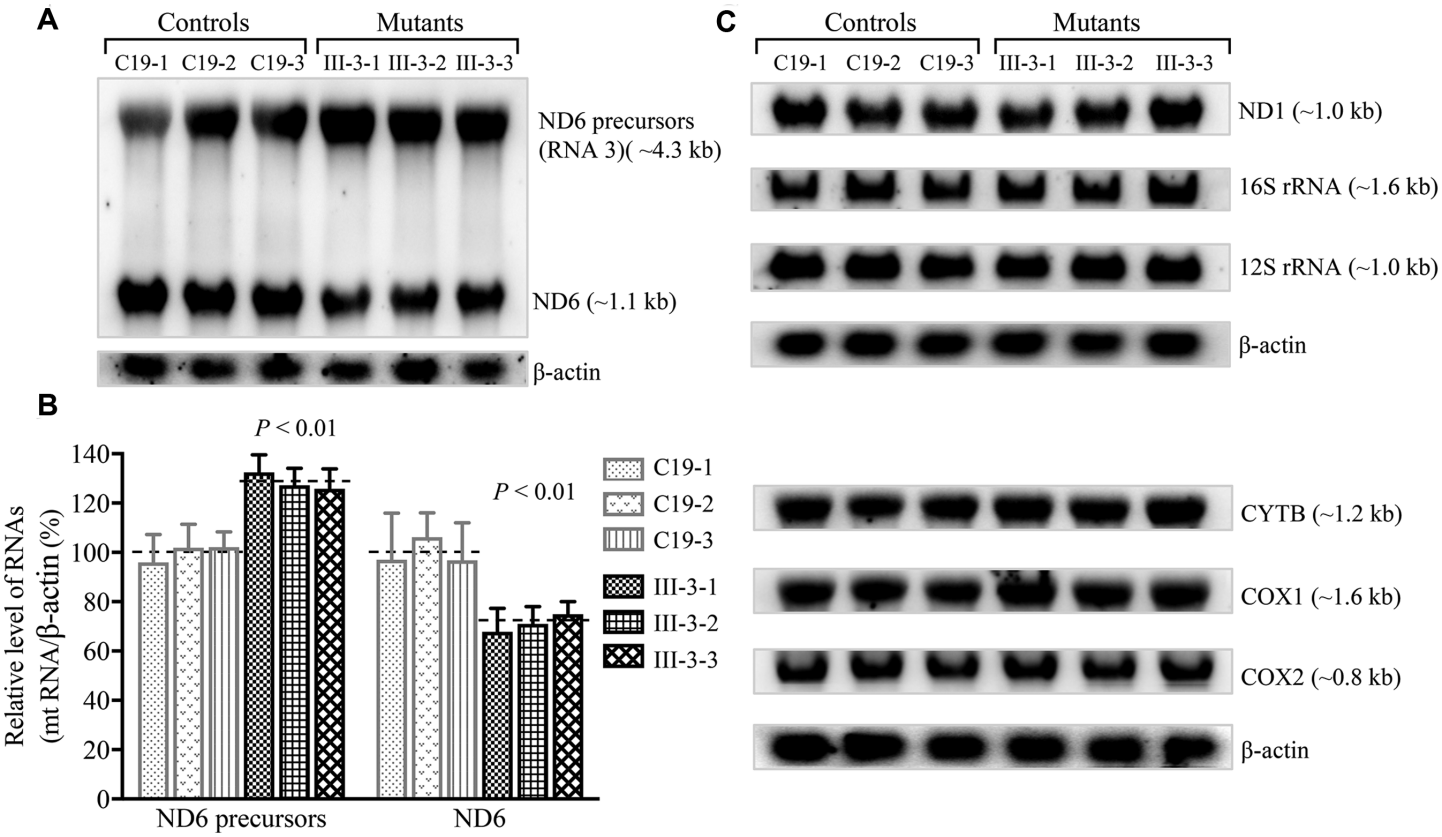
Northern blot analysis of mitochondrial RNAs. (**A, C**) Eight microgram of total cellular RNA from various mutant and control cybrids were electrophoresed through a 1.5% agarose‐formaldehyde gel, transferred onto a positively charged membrane and hybridized with DIG-labeled RNA probes for ND6 from L-strand, ND1, COX1, COX2, CYTB, 12S rRNA, 16S rRNA from H-strand, and *β*-actin as a control, respectively. (**B**) Average relative levels of ND6 mRNA and ND6 precursors per cell were normalized to the average level per cell of *β*-actin in three control cell lines and three mutant cell lines. The values for the latter are expressed as percentages of the average values for the control cell lines. Five independent determinations were used in the calculations. Graph details and symbols are explained in the legend to Figure 3.

We then tested whether the m.4401A>G mutation led to the aberrant processing of ND6 mRNA and accumulation of longer and uncleaved precursors. RNA transfer hybridization experiments were performed with total cellular RNAs from various mutant and control cybrids, using a set of DIG-labeled RNA probes: ND6 from L-strand transcript, ND1, COX1, COX2, CYTB, 12S rRNA, 16S rRNA from H-strand transcript and *β*-actin as a control, respectively. These experiments using ND6 probe revealed the presence of RNA species with ∼1.1 and ∼4.3 kb long, respectively, as shown in Figure 4A for mutant and control cell lines. The 4.3-kb RNA could conceivably be a precursor (RNA3) of ND6 mRNA (5,7,8) (Figure 1B). The ∼1.1-kb RNA was equal in size to the ND6 mRNA in mouse (1.15 kb) (54) and rat cells (1.1 kb) (55). Thus, ∼1.1-kb RNA is presumably human ND6 mRNA, comprising of 525-nt coding sequence and ∼600-nt of 3′ untranslated sequence (Figure 1B) (5). To verify the putative ND6 mRNA, three cDNA fragments [525 bp (coding sequence at positions 14149–14673), 697 bp (at positions13977–14673), 1,096 bp (at positions 13578–14673)] from the control cell line C19 and mutant cell line III-3 were analyzed by Sanger sequence after cDNA synthesis and PCR amplification (Supplemental Figure S5A and B). Indeed, all three fragments contained the identical 525 bp ND6 coding sequence, while 697 bp and 1,096 bp fragments encompassed the 3′ untranslated sequences. This result confirmed that ∼1.1 kb RNA was indeed ND6 mRNA. As shown in Figure 4A, the mutant cell lines exhibited the reduced levels of ND6 mRNA but accumulated precursors of ND6, as compared with those in the control cell lines. The average levels of ND6 mRNA and its precursors in three mutant cybrids, normalized with respect to those of β-actin mRNA, were 71.2% and 128.5% of those in the average values of three control cell lines, respectively (*P* < 0.01) (Figure 4B). However, the levels of ND1, COX1, COX2, CYTB, 12S rRNA, 16S rRNA and ND1 and 16S rRNA precursors [comprising of 16S rRNA, tRNA^Leu(UUR)^ and ND1] (8,11,27) from H-strand transcripts in mutant cell lines, normalized with respect to those of β-actin mRNA, were comparable with those in the control cell lines (Figure 4C, Supplemental Figure S5C). These results demonstrated that the m.4401A>G mutation perturbed the processing of ND6 mRNA and led to the accumulating precursors from the L-strand transcript but did not affect the processing of mRNAs and rRNAs from the H-strand transcripts.

### Mitochondrial translation defects

**Figure 5.**
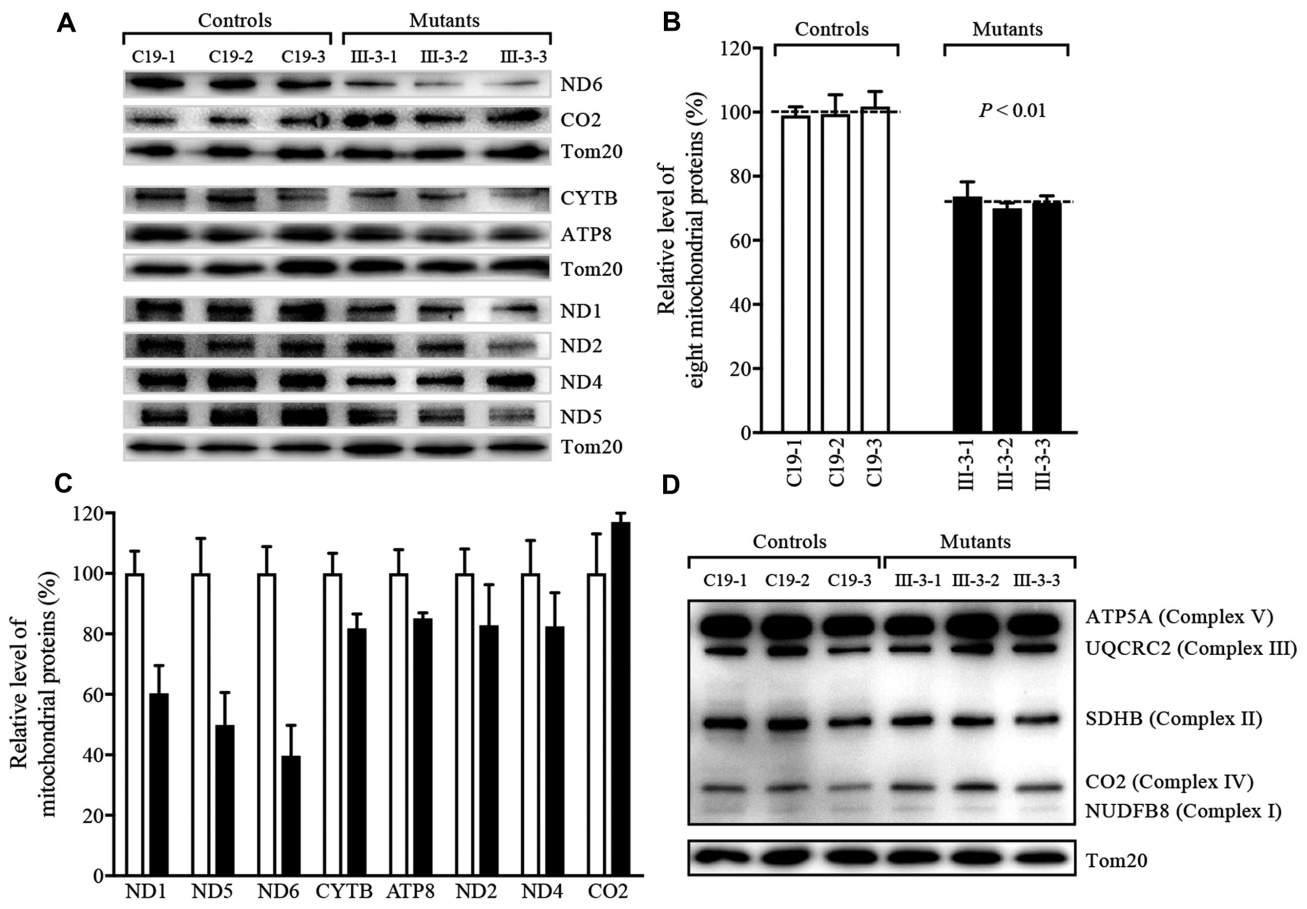
Western blot analysis of mitochondrial proteins. (**A**) Analysis of mtDNA encoding proteins. Five micrograms of total mitochondrial proteins from various cell lines were electrophoresed through a denaturing polyacrylamide gel, electroblotted and hybridized with antibodies specific for ND1, ND2, ND4, ND5, ND6, CO2, CYTB and ATP8 and with Tom20 as a loading control, respectively. (**B**) Quantification of total mitochondrial protein levels. The average levels of 8 mitochondrial proteins in mutant and control cell lines were determined as described elsewhere (29,43). (**C**) Quantification of 8 polypeptides. The levels of ND1, ND2, ND4, ND5, ND6, CO2, CYTB and ATP8 in mutant and control cell lines were determined as described elsewhere (29,43). (**D**) Analysis of five OXPHOS subunits encoded by mtDNA and nuclear genes. Five micrograms of total mitochondrial proteins from various cell lines were electrophoresed through a denaturing polyacrylamide gel, electroblotted and hybridized with antibody cocktail specific for subunits (ATP5A, UQCRC2, SDHB, CO2 and NDUFB8) of each OXPHOS complex and with TOM20 as a loading control. Graph details and symbols are explained in the legend to Figure 3.

To investigate whether the m.4401A>G mutation impaired mitochondrial translation, a Western blot analysis was carried out to examine the levels of 8 mtDNA encoded polypeptides: [ND1, ND2, ND4, ND5 and ND6 (subunits 1, 2, 4, 5 and 6 of NADH dehydrogenase), CO2 (subunit II of cytochrome c oxidase); CYTB (apocytochrome *b*) and ATP8 (subunit 8 of H^+^-ATPase)] in mutant and control cybrid cell lines with a nuclear encoding mitochondrial protein Tom20 as loading control. As shown in Figure 5A, the mutant cell lines exhibited the variable decrease in the levels of ND1, ND2, ND4, ND5, ND6, CYTB and ATP8 but the mild increase in the levels of CO2. The average overall levels of 8 mitochondrial translation products in the three mutant cybrid cell lines were 75% (*P* <0.01), relative to the mean value measured in three control cybrid cell lines (Figure 5B). As shown in Figure 5C, ND1, ND2, ND4, ND5, ND6, CO2, CYTB and ATP8 in the mutant cell lines were 60%, 83%, 82%, 50%, 40%, 117%, 81% and 85% of the average values of control cell lines, respectively.

We then examined the levels of five subunits of OXPHOS complexes in control and mutant cybrids by Western blot analysis using the total OXPHOS human antibodies cocktail containing antibodies for mtDNA encoded subunit CO2 of cytochrome c oxidase and four other polypeptides (NDUFB8 of NADH:ubiquinone oxidoreductase; SDHB of succinate ubiquinone oxidoreductase; UQCRC2 of ubiquinol cytochrome *c* reductase and ATP5A of H^+^-ATPase) encoded by nuclear genes. As shown in Figure 5D, the levels of CO2, NDUFB8, SDHB, UQCRC2 and ATP5A in mutant cybrid cell lines were comparable with those in control cybrid cell lines.

### Respiration deficiency

**Figure 6.**
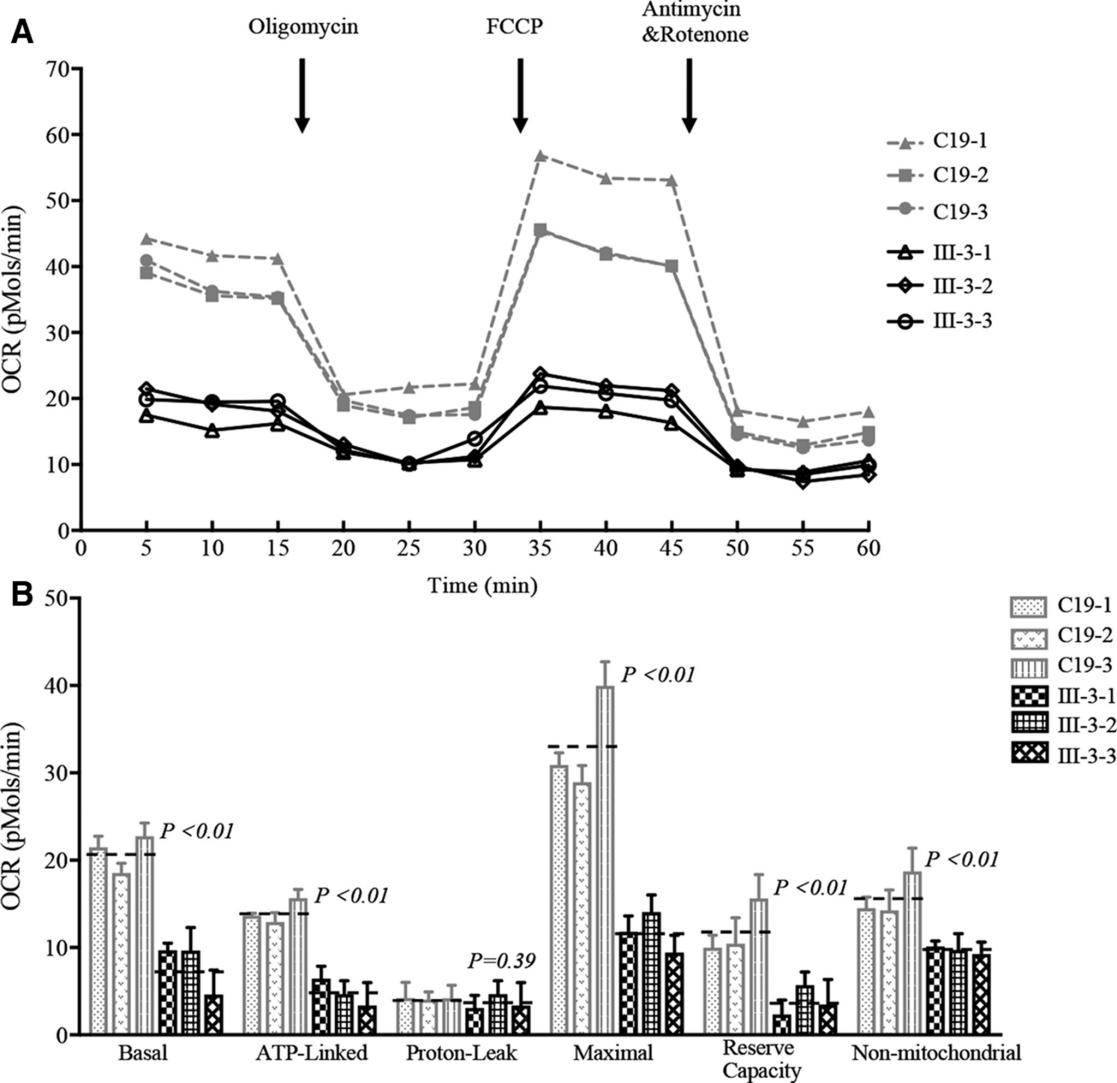
Respiration assays. (A) An analysis of O_2_ consumption in the various cell lines using different inhibitors. The rates of O_2_ (OCR) were first measured on 1 × 10^4^ cells of each cell line under basal condition and then sequentially added to oligomycin (1.5 μM), carbonyl cyanide p-(trifluoromethoxy) phenylhydrazone(FCCP) (0.15 μM), rotenone (1 μM) and antimycin A (5 μM) at indicated times to determine different parameters of mitochondrial functions. (B) Graphs presented the ATP-linked OCR, proton leak OCR, maximal OCR, reserve capacity and non-mitochondrial OCR in mutant and control cell lines. Non-mitochondrial OCR was determined as the OCR after rotenone/antimycinA treatment. Basal OCR was determined as OCR before oligomycin minus OCR after rotenone/antimycin A. ATP-linked OCR was determined as OCR before oligomycin minus OCR after oligomycin. Proton leak was determined as Basal OCR minus ATP-linked OCR. Maximal was determined as the OCR after FCCP minus non-mitochondrial OCR. Reserve Capacity was defined as the difference between Maximal OCR after FCCP minus Basal OCR. OCR values were expressed in picomoles of oxygen/minute/microgram of protein. The average values of 3 determinations for each cell line were shown. Graph details and symbols are explained in the legend to Figure 3.

To evaluate if the impairment of translation caused by the m.4401A>G mutation affects oxidative phosphorylation, we measured the oxygen consumption rate (OCR) of various mutant and control cybrid cell lines using a Seahorse Bioscience XF-96 Extracellular Flux Analyzer (43,46). As shown in Figure 6, the average basal OCRs in three mutant cybrids were 38% (*P* < 0.01) of the mean values measured in three control cybrid cell lines. To further investigate which of the enzyme complexes of the respiratory chain was affected in the mutant cybrid cell lines, OCR was measured after the sequential addition of oligomycin, FCCP, antimycin A and rotenone (56,57). The difference between the basal OCR and the drug-insensitive OCR resulted in the amount of ATP-linked OCR, proton leak OCR, maximal OCR, reserve capacity OCR and non-mitochondrial OCR. As shown in Figure 6B, the ATP linked OCR, proton leak OCR, maximal OCR, reserve capacity OCR and non-mitochondrial OCR in mutant cybrid cell lines were 34% (*P* < 0.01), 86% (*P* = 0.42), 36% (*P* < 0.01), 32% (*P* < 0.01) and 61% (*P* < 0.01) relative to the mean value measured in the control cybrid cell lines, respectively.

### Decrease in mitochondrial membrane potential

**Figure 7.**
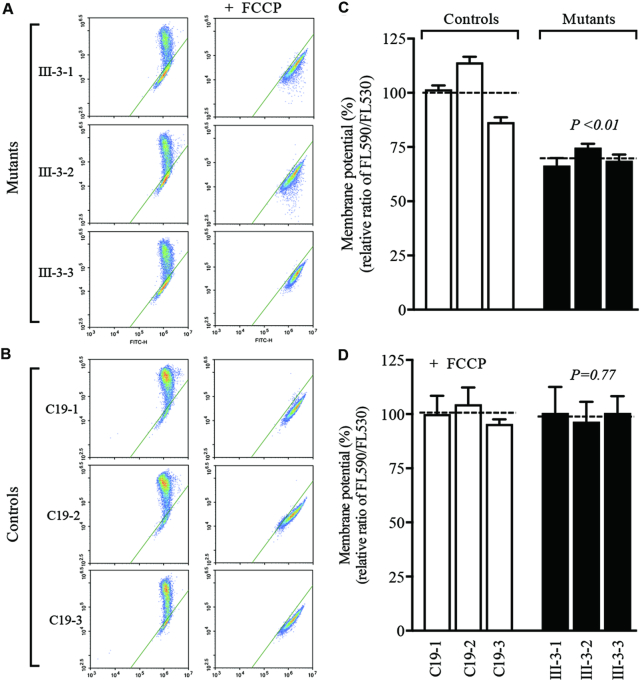
Mitochondrial membrane potential analysis. Mitochondrial membrane potential (ΔΨm) was measured in three mutant and three control cell lines using a fluorescence probe JC-10 assay system. The ratio of fluorescence intensities Ex/Em = 488/570 and 488/520 nm (FL570/FL520) were recorded to delineate the ΔΨm level of each sample. Represented flow cytometry images of mutant and control cell lines without (**A**) and with (**B**) 10 μM of FCCP. Relative ratio of JC-10 fluorescence intensities at Ex/Em = 488/570 and 488/520 nm in absence (**C**) and presence (**D**) of 10 μM of FCCP. The average of three to five determinations for each cell line is shown. Graph details and symbols are explained in the legend to Figure 3.

The mitochondrial membrane potential (ΔΨm) generated by proton pumps (Complexes I, III and IV) is the central bioenergetic parameter that controls respiratory rate, ATP synthesis and the generation of reactive oxygen species (47). The mitochondrial membrane potentials (ΔΨm) of three mutant and three control cell lines were measured through the fluorescence probe JC-10 assay system. As illustrated in Figure 7, the average levels of the ΔΨm in three mutant cybrids carrying the m.4401A>G mutation ranged from 67% to 74%, with an average of 70% (*P* < 0.01) of the mean value measured in three control cybrids. Conversely, the levels of ΔΨm in three mutant cybrids in the presence of FCCP were comparable with those measured in three control cybrid cell lines.

### Increasing production of mitochondrial ROS

**Figure 8.**
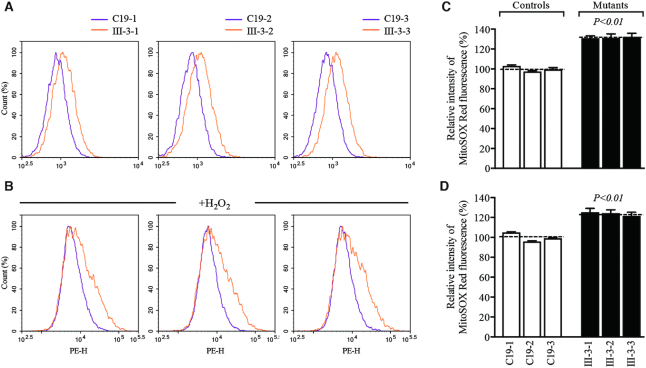
Measurement of mitochondrial ROS. Ratio of geometric mean intensity between levels of the ROS generation in the vital cells with or without H_2_O_2_ stimulation. The rates of mitochondrial ROS production in three mutant cybrid cell lines and three control cell lines were analyzed by Novocyte flow cytometer (ACEA Biosciences) system using MitoSOX Red Mitochondrial Superoxide Indicator. Flow cytometry histogram showing MitoSOX-Red fluorescence of three control cybrids (purple) and three mutant cybrids (orange) without (**A**) or with (**B**) H_2_O_2_ stimulation. The relative ratios of fluorescence intensity were calculated in the absence (**C**) and presence (**D**) of H_2_O_2_. Graph details and symbols are explained in the legend to Figure 3.

Excess productions of ROS caused by mitochondrial dysfunctions have been linked to the pathology of hypertension (58,59). To assess if the m.4401A>G mutation elevated the production of mitochondrial ROS, the levels of mitochondrial ROS generation in three mutant cybrids cell lines carrying the m.4401A>G mutation and three control cybrid cell lines lacking the mutation were measured using MitoSOX assay via flow cytometry under normal conditions and then following H_2_O_2_ stimulation (29,48). Geometric mean intensity was recorded to measure the production rate of ROS of each sample. As shown in Figure 8A and B, the levels of ROS generation in the mutant cybrid cell lines carrying the m.4401A>G mutation ranged from 130.9% to 132.2%, with an average of 131.4% (*P* < 0.01) of the mean value measured in control cybrid cell lines under unstimulated conditions. As illustrated in Figure 8C and D, the levels of ROS generation in three mutant cybrid cell lines varied from 121.6% to 125.3%, with an average of 123.8% (*P* < 0.01) of the mean values measured in three control cybrid cell lines under stimulation conditions.

### Promoting autophagy

**Figure 9.**
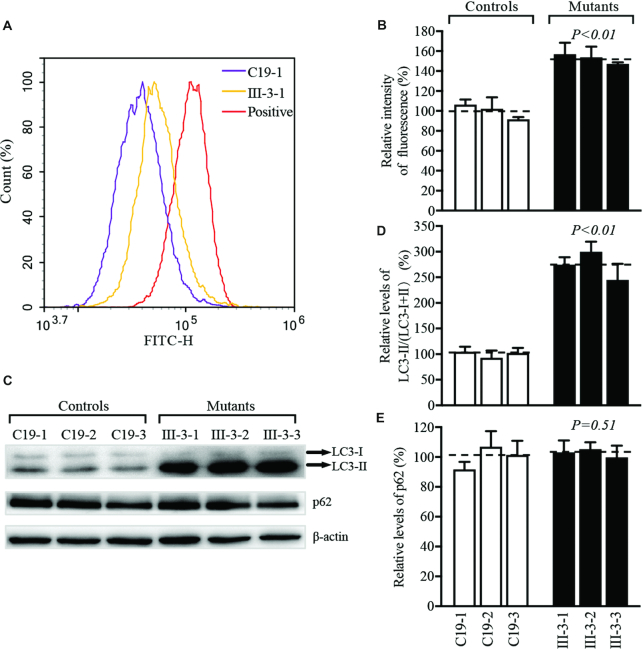
Analysis of autophagy. (**A**) Fluorescence histograms of representative mutant (III-3-1) and control (C19-1) cybrids measured using CYTO-ID^®^ Autophagy Detection Kit. Cells were incubated with DMEM in the absence and presence of rapamycin (inducers of autophagy) and chloroquine (lysosomal inhibitor) at 37°C for 18 h, added to CYTO-ID®-Green dye and analyzed using a Novocyte flow cytometer (ACEA Biosciences). (**B**) The relative ratios of fluorescence intensity. (**C**) Western blot analysis of proteins LC3-I/II and p62. Twenty micrograms of total cellular proteins from various mutant and control cell lines were electrophoresed, electroblotted and hybridized with LC3, p62 and with β-actin as a loading control. (**D, E**) Quantification of autophagy markers LC3-I/II and p62 in mutant and control cell lines were determined as described elsewhere.^36^ The average of three determinations for each cell line were shown. Graph details and symbols are explained in the legend to Figure 3.

To investigate if the increasing production of mitochondrial ROS caused by the m.4401A>G mutation regulated the autophagy, the autophagic states of various mutant and control cell lines were analyzed using both fluorescence-based cytometry and Western-blotting assays. First, CYTO-ID^®^ Autophagy Detection Kits were used with flow cytometry to examine the degree of autophagy of mutant and control cell lines (49,50). As shown in Figure 9A, a significant shift in the fluorescence peak to high intensity occurred in the mutant cell lines, as compared with those in controls. The levels of autophagy in three mutant cells varied from 147.2% to 156.8%, with an average of 152.6% (*P* < 0.01) of the mean values measured in three control cybrid cell lines (Figure 9B). We then performed Western blot analysis using two markers: microtubule-associated protein 1A/1B light chain 3B (LC3) and sequestosome 1 (SQSTM1/p62) (60,61). During autophagy, the cytoplasmic form (LC3-I) is processed into a cleaved and lipidated membrane-bound form (LC3-II), which is essential for membrane biogenesis and closure of the membrane. LC3-II is recleaved by cysteine protease (Atg4B) following completion of the autophagosome and recycled (62). SQSTM1/p62, one of the best-known autophagic substrates, interacts with LC3 to ensure the selective delivery of these proteins into the autophagosome (60). As shown in Figure 9C and D, the levels of LC3-II/(LC3-I+II) in three mutant cybrids bearing the m.4401A>G mutation were markedly increased, with an average of 273.1% (*P* < 0.01) of the mean values measured in three control cell lines lacking the mutation. However, the levels of p62 in mutant cell lines carrying the m.4401A>G mutation were comparable with those in three control cell lines lacking the mutation (Figure 9C and E). These data suggested that the m.4401A>G mutation promoted the autophagy in the mutant cybrids.

### Reduced capacity of wound healing

**Figure 10.**
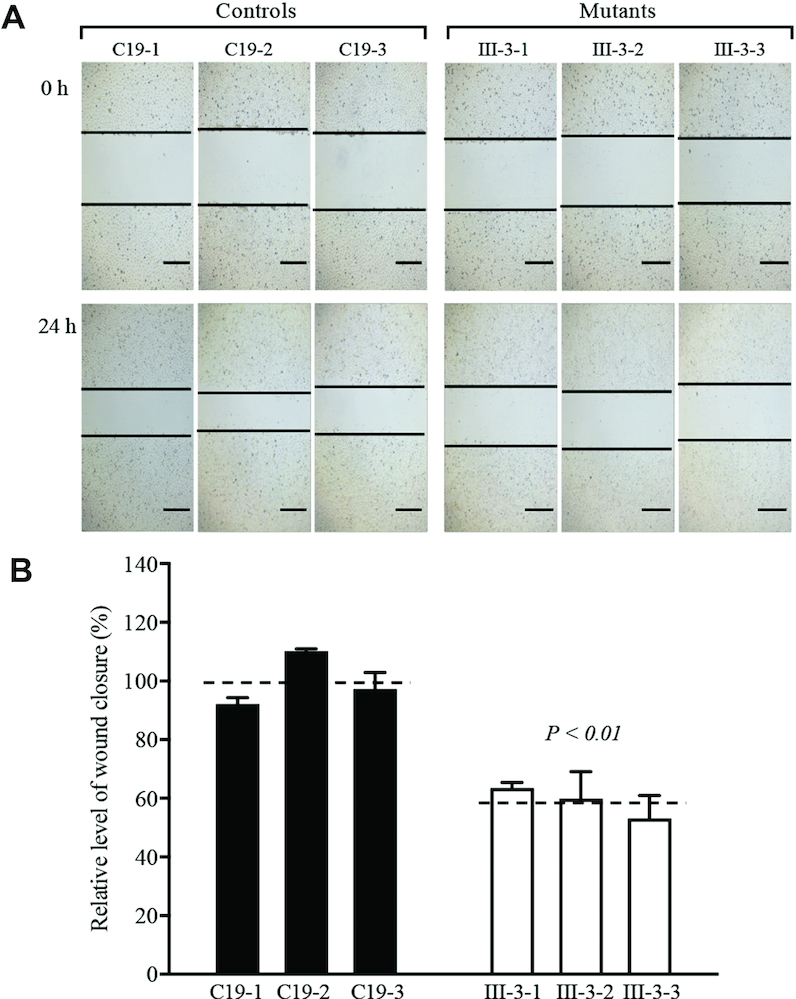
Wound healing assay. (**A**) Representative images of wound healing assay for various control and mutant cybrid cell lines. Photographs were taken directly immediately after scratch and 24 h after wounding. The outlines show the gap area of the wounds. Scale bars, 100 μm. (**B**) Quantification of wound healing rates. Quantitative measurement of cell migration was performed at 24 h after wounding as described previously (33). The calculations were based on 3–4 independent determinations in each cell line. Graph details and symbols are explained in the legend to Figure 3.

To examine the effect of m.4401A>G mutation-induced alterations on angiogenesis and wound regeneration, the wound healing assays were carried out in various mutant and control cybrids with live-cell microscopy (33,51,63). For this purpose, various cybrids were wounded with a scratch and incubated with serum free medium for 24 h to impair healing and then visualized by an optical microscopy. As showed in Figure 10A, wound healing cell migrations in the mutant cybrid cell lines were significantly lower than those in control cybrid cell lines after culture for 24 h after wounding. As showed in Figure 10B, the levels of wound closure in three mutant cybrid lines ranged from 53.1% to 63.5%, with an average of 58.8% (*P* < 0.01), compared with the average values of three control cybrid lines.

### Weaken cellular angiogenesis

**Figure 11.**
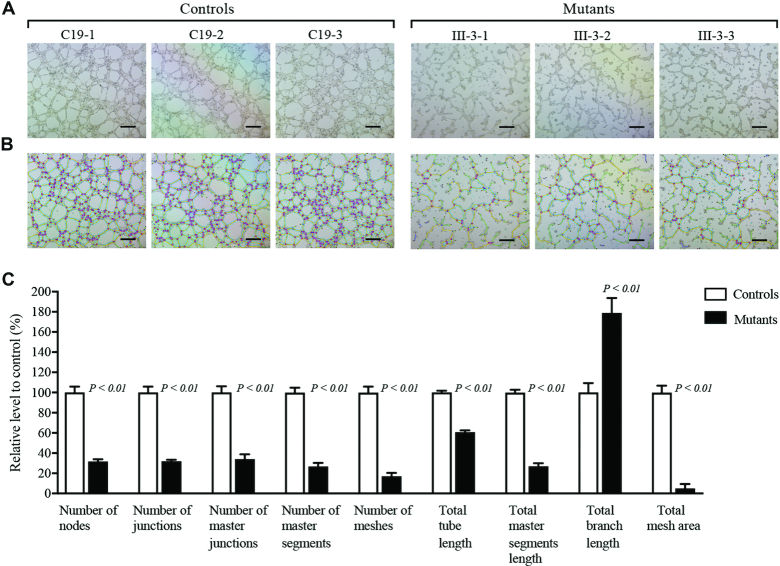
Tube formation assay. Cells (3 × 10^5^) from various control and mutant cybrid cells were grown and resuspended with medium and loaded on top of Matrigel. Following incubation at 37°C for 10 hours, each well was analyzed directly for tube formation under an inverted microscope. (**A**) Representative light photomicrographs of tube formation for three control cybrids and three mutant cybrids after plating onto Matrigel for 10 h. (**B**) The photomicrographs were analyzed using Angiogenesis Analyzer (ImageJ), different structures of the tubule network are labeled with different colors representing its own feature: master segments (orange), meshes (sky blue), nodes surrounded by junction symbol (red surrounded by blue) and branches (green). (**C**) Quantitative analysis of specific parameters of capillary tube formation with the Angiogenesis Analyzer for ImageJ. The parameters include number of nodes, number of junctions, number of master junctions, number of master segments, number of meshes, total tube length, total master segments length, total branch length, and total mesh area. Data represent an average of nine fields of each cell line. Scale bars, 500 μm. Graph details and symbols are explained in the legend to Figure 3.

The effect of m.4401A>G mutation on angiogenesis was further evaluated in various mutant and control cell lines by the tube formation assay (52,53). The various cell lines were cultured in the presence of growth factor-reduced Matrigel, an extract of endothelial basement membrane, for 16 h, to induce the differentiation and tube-like structure formation. Cells gradually stretched, and connected each other into cords and network structure, forming luminal structures of various sizes and shapes after loading on the top of Matrigel (Figure 11A). The captured images were analyzed by ImageJ with the Angiogenesis Analyzer plugin to quantify different parameters, such as master segments (orange), meshes (sky blue), nodes surrounded by junction symbol (red surrounded by blue) and branches (green) (Figure 11B). As illustrated in Figure 11C, the numbers of nodes, junctions, master junction, master segments and meshes, the total tube length, master segments length, branch length and the total mesh area in three mutant cell lines were 32%, 32%, 34%, 27%, 17%, 61%, 27%, 179% and 5%, relative to the mean values measured in three control cell lines, respectively. The alterations in tube formation indicated that the m.4401A>G mutation affected cellular angiogenesis.

## DISCUSSION

Mitochondrial tRNA mutations are the important causes of hypertension, accounting for 3.9% cases of 2070 Han Chinese hypertensive subjects (6). The tissue-specific manifestation of hypertension-associated tRNA mutations remains elusively. Using HUVEC-derived cybrids under the constant nuclear and mitochondrial backgrounds, we demonstrated the profound impact of hypertension-associated m.4401A>G mutation on mitochondrial function contributing to the pathological process of hypertension. In fact, the m.4401A>G mutation lies in the spacer immediately to the 5′ ends of the tRNA^Met^ at the H-strand transcript and tRNA^Gln^ at the L-strand transcript (6,30). Therefore, it was anticipated that the primary defects arising from the m.4401A>G mutation were the aberrant 5′ end processing of tRNA^Met^ and tRNA^Gln^ precursors, catalyzed by RNase P. In this study, the *in vitro* processing analysis revealed marked reductions in the efficiency of the 5′ end processing of tRNA^Met^ and tRNA^Gln^ precursors carrying the m.4401A>G mutation, catalyzed by RNase P. By contrast, the m.4263A>G or m.5655A>G mutation caused relatively lower decreases in the efficiency of the 5′ end processing of tRNA^Ile^ or tRNA^Ala^ precursors carrying the mtDNA mutation(s), respectively (28,29). The aberrant processing of tRNA^Met^ and tRNA^Gln^ precursors were further supported by the observations that HUVEC-derived cybrids and parental lymphoblastoid cell lines bearing the m.4401A>G mutation exhibited significant reductions in the steady-state levels of tRNA^Met^ and tRNA^Gln^ (30). However, the mechanisms involved in tRNA processing defects arising from the m.4401A>G mutation differed for the L-strand and H-strand polycistronic transcripts. On the H-strand transcript, there were almost similar levels of other matured 13 tRNAs such as tRNA^Leu(UUR)^, 12S rRNA, 16S rRNA, mRNAs and their precursors between the mutant and control cell lines. The processing of the precursor H-strand transcripts, excised by RNase P and RNase Z, referred to as ‘the tRNA punctuation model’, may undergo simultaneously as the transcriptions of HSP2 proceeds (7,8,14,64). As a result, the aberrant tRNA^Met^ 5′ end processing caused by the m.4401A>G mutation did not affect the processing of other 13 tRNAs including tRNA^Leu(UUR)^, tRNA^Lys^, 12 mRNAs such as ND1 and COXII, 12S rRNA and 16S rRNA which are co-transcribed from the L-strand mtDNA (7,11–14). Conversely, the m.4401A>G mutation not only impaired the processing of all 8 tRNAs and ND6 but also caused the accumulation of longer and uncleaved precursors from the L-strand transcript. These were evidenced by lower levels of ND6 mRNA, tRNA^Ala^, tRNA^Asn^, tRNA^Cys^, tRNA^Tyr^, tRNA^Ser(UCN)^, tRNA^Glu^, and tRNA^Pro^ and the accumulation of longer and uncleaved precursors in the mutant cybrids, as compared with those in control cybrids. In fact, the deafness-associated m.7445A>G mutation in a spacer immediately to the 3′ end of tRNA^Ser(UCN)^ not only altered the 3′ end processing of tRNA^Ser(UCN)^ precursor but also had long-range effects on ND6 expression (5,23). These observations suggested that the processing of L-strand polycistronic transcripts likely initiates by cleavage of the 3′ end and 5′ end of tRNA^Gln^ by RNase P and RNase Z until the transcriptions of LSP terminates. These data demonstrated the asymmetrical processing mechanisms of H-strand and L-strand polycistronic transcripts.

The shortages of tRNA^Met^, all eight tRNAs and ND6 mRNAs from L-strand transcript resulted in the impairment of mitochondrial translation. In this study, ∼25% reduction in the overall levels of eight mtDNA encoding proteins were observed in mutant cybrids carrying the m.4401A>G mutation, in agreement with ∼30% reduction in the rates of mitochondrial translation in lymphoblastoid cell lines carrying the m.4401A>G mutation (30). However, there were variable decreases in the levels of ND1, ND3, ND4, ND5, ND6, CYTB and ATP8 in the mutant cell lines. Especially, mutant cybrids bearing the m.4401A>G mutation exhibited marked reduction (60%) in the level of ND6 but mildly increased level (17%) of CO_2_. The marked decrease of ND6 level likely resulted from both shortage of nine tRNAs and lower levels of ND6 mRNA, caused by the m.4401A>G mutation, as in the case of those in cell lines carrying the m.7445A>G mutation (5). However, the levels of four subunits of OXPHOS complexes (NDUFB8, SDHB, UQCRC2 and ATP5A) encoded by nuclear genes were not changed in the mutant cybrids bearing the m.4401A>G mutation, indicating the negligible effect of m.4401A>G mutation on the stability of OXPHOS complexes. The impaired synthesis of these mtDNA encoding 13 polypeptides caused the dysfunctions of mitochondrial electron transport chain (64–67). Strikingly, the rates of O_2_ consumption were reduced much lower in HUVEC derived cybrids cell lines than those in lymphoblastoid cell lines. In particular, the HUVEC derived cybrids carrying the m.4401A>G mutation displayed markedly reduced rates in the basal OCR (62%), ATP-linked OCR (66%), maximal OCR (64%) and reserve capacity (68%), in contrast with 20–23% reductions in the rates of total O_2_ consumption, complex I, complex III and complex IV-derived O_2_ consumption observed in the lymphoblastoid cell lines derived from a Chinese family carrying the m.4401A>G mutation (30). These data were correlated with ∼45% and 59% reductions in the levels of tRNA^Met^ and tRNA^Gln^ in the mutant HUVEC-derived cybrids, and ∼30% reductions in the steady state levels of these tRNAs in the mutant lymphoblastoid cell lines (30). These discrepancies may be attributed to the cell/tissue specific effects on mitochondrial functions, especially tRNA metabolism and oxidative phosphorylation (68–71). Dysfunctions in the ETC caused by the m.4401A>G mutation diminished the ATP production, mitochondrial membrane potentials, increased the generation of ROS and subsequently disturbed the redox balance by altering the NAD/NADH ratio (57,72,73). The impairment of oxidative phosphorylation and mitochondrial membrane potential yielded the increasing production of ROS in mitochondria, which is the major site of ROS production (10,58,74,75). The overproduction of mitochondrial ROS played a key role in the pathogenesis of cardiovascular disease, especially worsening mitochondrial abnormalities and endothelial dysfunction (76–80). Alterations in OXPHOS and mitochondrial membrane potential as well as overproduction of mitochondrial ROS affected the mitophagic removal of damaged mitochondria (81,82). In this study, markedly increased levels of LC3 but almost no changes in the levels of p62 in cybrids carrying the m.4401A>G mutation suggested a general increase in the capacity of the mutant cells to generate autophagosomes (83), in contrast with the reduced levels of LC3 in mutant cell lines carrying the LHON-associated ND5 12338T>C mutation (36). These indicated that the m.4401A>G mutation affected the autophagy by elevation of the autophagic degradation of ubiquitinated proteins.

The m.4401A>G mutation-induced mitochondrial dysfunctions, including decreased respiration, ATP production, and increased production of ROS led to the profound impacts on the vital functions of endothelial cells (74,84–87). In this present study, the specific effects of m.4401A>G mutation on angiogenesis were evidenced by aberrant wound regeneration and weaken cellular angiogenesis in HUVECs-derived cybrids. Especially, the mutant cybrid cell lines exhibited lower wound healing cell migration than those in control cybrid cell lines. Furthermore, various parameters for angiogenesis, including the number of nodes, junctions, master junction, master segments and meshes, the lengths of total tube, master segments, total mesh area in the HUVECs-derived mutant cybrid lines displayed significant decreases as compared to those in control cybrid lines. These dysfunctions of endothelial cells demonstrated that the m.4401A>G mutation-induced mitochondrial dysfunctions contributed to the development of hypertension. It is worthwhile to note that aberrant wound regeneration and weaken cellular angiogenesis were also observed in the HUVECs-derived cybrids coronary artery disease assocm.15927G>A mutation (33). The clinical heterogeneity manifested by these similar biochemical defects is likely attributed to the involvement of nuclear genetic and epigenetic factors (10,65,68,88).

In summary, our findings demonstrated the pathogenic mechanism leading to an impaired oxidative phosphorylation in HUVECs-derived cybrid cell lines carrying the hypertension-associated m.4401A>G mutation. The m.4401A>G mutation perturbed the 5′ end processing of tRNA^Met^ and tRNA^Gln^ precursors. The m.4401A>G mutation yielded the low level of tRNA^Met^ but did not affect the levels of other 13 tRNAs, 12 mRNAs 12S rRNA and 16S rRNA from the H-strand transcript. Conversely, the m.4401A>G mutation led to the significant decreases in the levels of all 8 tRNAs and ND6 but the accumulation of longer and uncleaved precursors from the L-strand transcript. These implicated the asymmetrical processing mechanisms of H-strand and L-strand polycistronic transcripts. The tRNA processing defects resulted in the impairing mitochondrial translation, respiratory deficiency, diminishing membrane potential, increasing production of reactive oxygen species and altering autophagy. Furthermore, the m.4401A>G mutation altered the angiogenesis, evidenced by aberrant wound regeneration and weaken tube formation in mutant cybrids. Our findings provide new insights into the pathophysiology of hypertension arising from mitochondrial tRNA processing defects.

## Supplementary Material

gkz742_Supplemental_File
