# Supplementary material for: Hypertension-associated mitochondrial DNA 4401A>G mutation caused the aberrant processing of tRNAMet, all 8 tRNAs and ND6 mRNA in the light-strand transcript
Source: Nucleic Acids Res. 2019 Aug 28;47(19):10340–56. doi: 10.1093/nar/gkz742 (PMC6821173; doi:10.1093/nar/gkz742)
Supplement: gkz742_Supplemental_File [file gkz742_supplemental_file.pdf]

SUPPLEMENTAL DATA

Supplemental Figure S1

Supplemental Figure S2

Supplemental Figure S3

Supplemental Figure S4

Supplemental Figure S5

Supplemental Table S1

Supplemental Table S2

Supplemental Figure S1

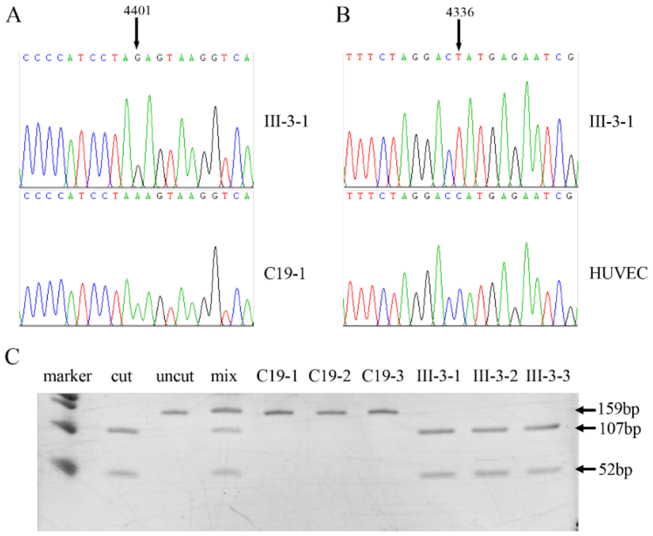

Supplemental Figure S1. Identification and quantification of the m.4401A>G and m.4336T>C mutations. (A) Sequence electropherograms of the mtDNA fragments covering position 4401 from the cybrid cell lines of an affected individual (III-3) and one genetically unrelated control individual (C19) respectively. An arrow indicates the location of the base changes at position 4401. (B) Sequence electropherograms of the mtDNA fragments covering position 4336 from the affected individual (III-3) and HUVECs respectively. An arrow indicates the location of the base changes at position 4336. (C) Quantification of the m.4401A>G mutation by PCR-RFLP. PCR products were digested with *BfaI* (4401) and analyzed by electrophoresis in a 10% polyacrylamide gel stained with ethidium bromide.

Supplemental Figure S2

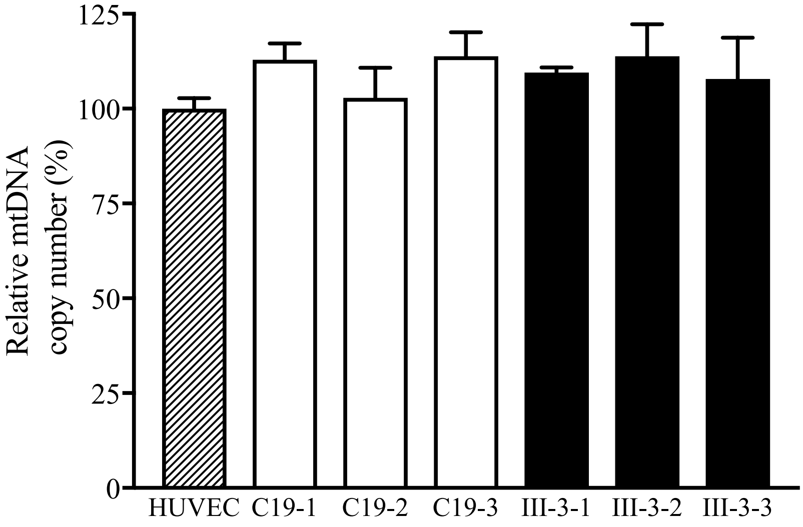

Supplemental Figure S2. Measurements of mtDNA copy numbers in cybrid cell lines. The mtDNA copy numbers were determined by comparing the ratio of mtDNA to nDNA (18S) by real-time quantitative PCR. The calculations were based on three independent determinations. The *error bars* indicate two standard errors of the means.

Supplemental Figure S3

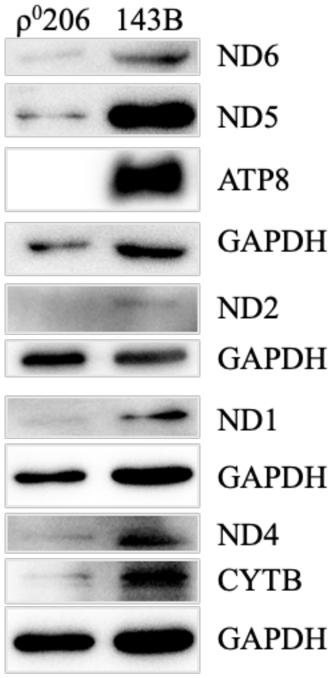

Figure S3. Western blot analysis of 7 mtDNA encoding proteins. Fifteen micrograms of total cellular proteins from 143B and $\rho^0$206 cell lines were electrophoresed through a denaturing polyacrylamide gel, electroblotted and hybridized with antibodies specific for ND1, ND2, ND4, ND5, ND6, CYTB and ATP8 and with GAPDH a loading control, respectively.

Supplemental Figure S4

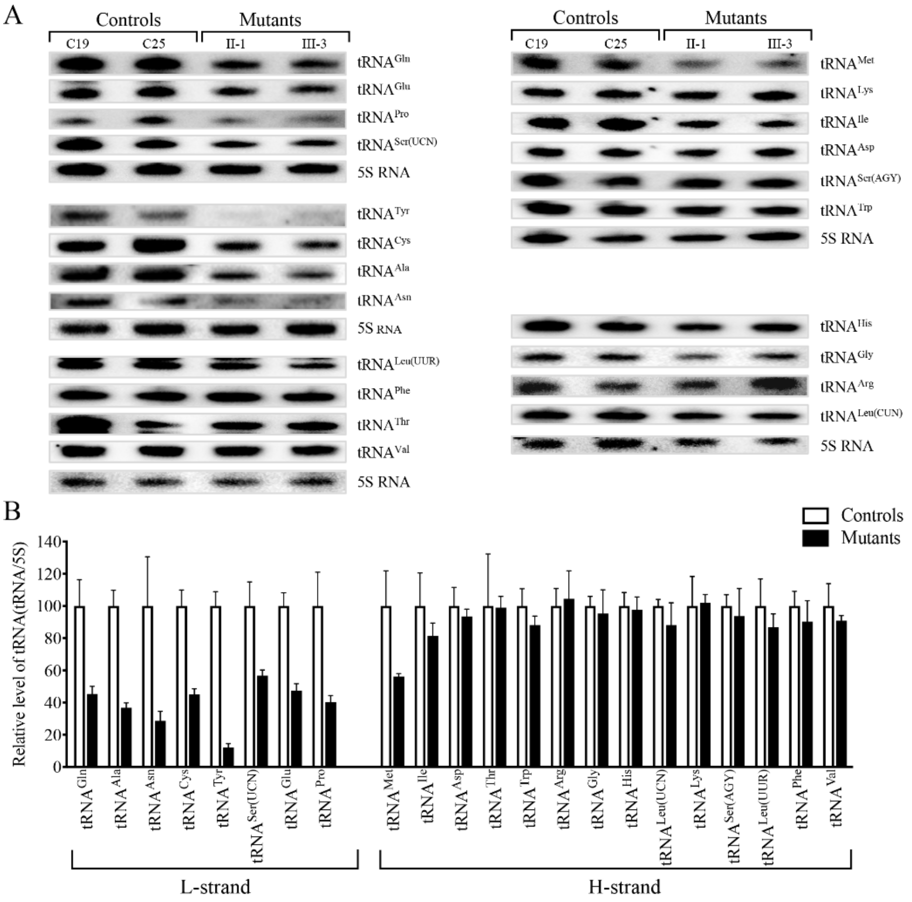

Figure S4. Northern blot analysis of mitochondrial tRNAs. (A) Equal amounts (2 μg) of total mitochondrial RNAs from the various lymphoblastoid cell lines were electrophoresed through a denaturing polyacrylamide gel, were electroblotted, and were hybridized with DIG-labeled oligonucleotide probes specific for $tRNA^{Gln}$, $tRNA^{Ala}$, $tRNA^{Asn}$, $tRNA^{Cys}$, $tRNA^{Tyr}$, $tRNA^{Ser(UCN)}$, $tRNA^{Glu}$, and $tRNA^{Pro}$ from the light (L)-strand transcription units, $tRNA^{Met}$, $tRNA^{Thr}$, $tRNA^{Trp}$, $tRNA^{Ile}$, $tRNA^{Leu(UUR)}$, $tRNA^{Leu(CUN)}$, $tRNA^{Lys}$, $tRNA^{Ser(AGY)}$, $tRNA^{His}$, $tRNA^{Gly}$, $tRNA^{Phe}$, $tRNA^{Asp}$, $tRNA^{Asn}$, and $tRNA^{Val}$ from the heavy (H)-strand transcription units, and 5S rRNA, respectively. (B) Quantification of the tRNA levels. Average relative each tRNA content per cell was normalized to the average content per cell of 5S rRNA in the control cell lines and in the mutant cell lines, respectively. The values for the latter are expressed as percentages of the average values for the control cell lines. The calculations were based on three independent determinations in each cell line. The error bars indicate two standard errors of the mean (SEM). *P* indicates the significance, according to the t-test, of the differences between mutant and control cell lines.

Supplemental Figure S5

A

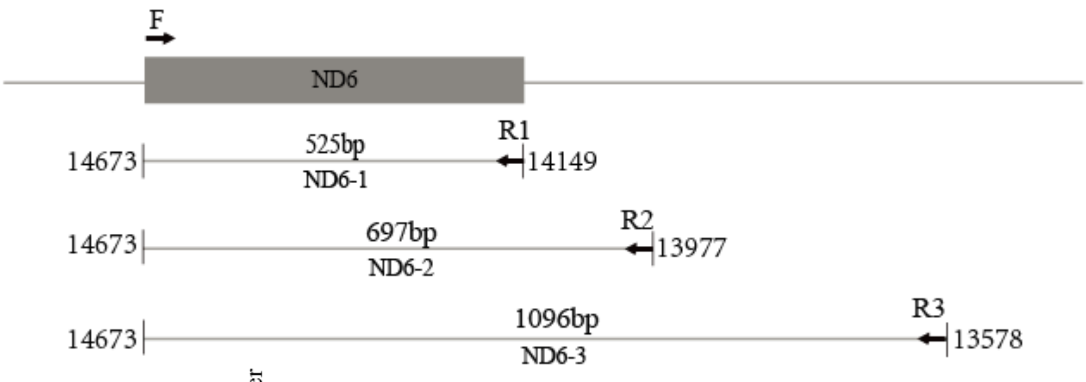

B

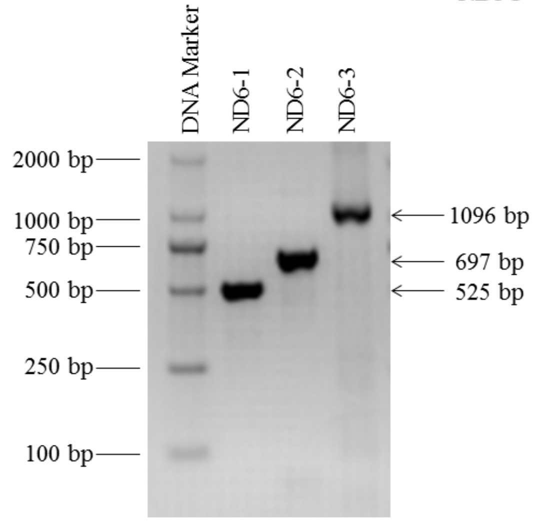

C

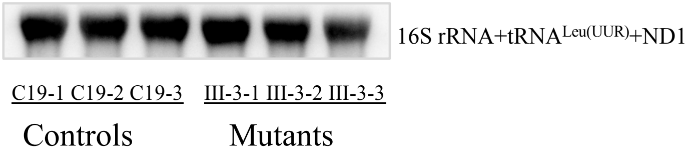

Figure S5. Identification of human ND6 cDNA and Northern blot analysis of ND1 precursor. (A) Schema for designing primers for amplification of various lengths of ND6 cDNAs. (B). Three ND6 cDNAs [525 bp (coding sequence at positions 14149-14673), 697 bp (at positions13977-14673), 1,095 bp (at positions 13578-14673)] from the control cell line C19 were analyzed by electrophoresis in a 1% agarose gel stained with ethidium bromide after cDNA synthesis and PCR amplification. Primers for RT-PCR amplification were as follows:
F: 5'-ATGATGTATGCTTTGTTTCTG-3', R1: 5'-CCTATTCCCCCGAGCAATCTC-3', R2: 5'-CCTGCCCCTACTCCTCCTAGACC-3', R3:5'CGCTACCTCCCTGACAAGCGCC-3'.
(C). Eight microgram of total cellular RNA from various mutant and control cybrids were electrophoresed through a 1.5% agarose-formaldehyde gel, transferred onto a positively charged membrane and hybridized with DIG-labeled RNA probe for ND1.

Supplemental Table S1. mtDNA variants in one Chinese hypertensive subject (IIII-3) and two Chinese control subjects (C19 and C25)

Gene	Position	Replacement	CRS*	C19	C25	III-3	Previously reported†
D-loop	73	A to G	A	G	G	G	Yes
	195	T to C	T	C			Yes
	199	A to G	A				Yes
	249	A to Del	A	Del A	Del A	Del A	Yes
	263	A to G	A	G	G		Yes
	310	T to TC	T	CTC	CTC	CTC	Yes
	489	T to C	T	C	C	C	Yes
	16129	G to A	G	A			Yes
	16145	G to A	G			A	Yes
	16223	C to T	C	T		T	Yes
	16298	T to C	T	C		C	Yes
	16327	C to T	C	T		T	Yes
	16519	T to C	T	C		C	Yes
12S RNA	750	A to G	A	G	G	G	Yes
	1438	A to G	A	G	G	G	Yes
16S RNA	1715	C to T	C	T	T		Yes
	2706	A to G	A	G	G	G	Yes
ND1	3552	T to A	T	A		A	Yes
non coding	4401	A to G	A			G	Yes
ND2	4715	A to G	A		G	G	Yes
	4769	A to G	A		G	G	Yes
	5262	G to A (Ala to Thr)	G			A	Yes
$tRNA^{Cys}$	5821	G to A	G			A	Yes
CO1	5993	C to T	C			T	Yes
	6026	G toA	G		A		
	6029	C to A	C	A			Yes
	6338	A to G	A			G	Yes
	6386	C to T	C			T	Yes
	7028	C to T	C	T	T	T	Yes
	7196	C to A	C	A	A	A	Yes
ATP6	8584	G to A (Ala toThr)	G		A	A	Yes
	8701	A to G (Thr to Ala)	A	G	G	G	Yes
	8860	A to G (Thr to Ala)	A	G	G	G	Yes
CO3	9540	T to C	T		C	C	Yes
	9545	A to G	A		G	G	Yes

ND3	10398	A to G (Thr to Ala)	A	G	G	G	Yes
	10400	C to T (Thr to Ala)	C	T	T	T	Yes
ND4	10873	T to C	T	C		C	Yes
	11447	G to A (Val to Met)	G			A	Yes
	11719	G to A	G	A			Yes
	11914	G to A	G	A		A	No
ND5	12672	A to G	A				Yes
	12705	C to T	C			T	Yes
	12858	C to T	T			T	Yes
	13263	A to G	A			G	Yes
ND6	14318	T to C (Asn to Ser)	T			C	Yes
Cytb	14783	T to C	T			C	Yes
	15043	G to A	G			A	Yes
	15301	G to A	G		A	A	Yes
	15487	A to T	A			T	Yes
	15833	C to T	C	T			Yes

*CRS: Cambridge reference sequence

† See the online mitochondrial genome database http://www.mitomap.org and http://www.genpat.uu.se/mtDB/

Supplemental Table 2. DIG-labeled oligodeoxynucleotide probes of 22 mitochondrial tRNA and 5S RNA.

RNA	Oligodeoxynucleotide sequence	Size (mer)
$tRNA^{Phe}$	5’-TGTTTATGGGGTGATGTGAGCCCGTCTAAA-3’	30
$tRNA^{Val}$	5’-TCAGAGCGGTCAAGTTAAGTTGAAATCTCC-3’	30
$tRNA^{Leu(UUR)}$	5’-AGAAGAGGAATTGAACCTCTGACTGTAAAG-3’	30
$tRNA^{Ile}$	5’-AGAAATAAGGGGGTTTAAGCTCCTATTATT-3’	30
$tRNA^{Gln}$	5’-AGGACTATGAGAATCGAACCCATCCCTGAG-3’	30
$tRNA^{Met}$	5’-TAGTACGGGAAGGGTATAACCAACATTTTC-3’	30
$tRNA^{Trp}$	5’-AGAAATTAAGTATTGCAACTTACTGAGGGC-3’	30
$tRNA^{Ala}$	5’-AGGACTGCAAAACCCCACTCTGCATCAACT-3’	30
$tRNA^{Asn}$	5’-CCAATGGGACTTAAACCCACAAACACTTAG-3’	30
$tRNA^{Cys}$	5’-CCCCGGCAGGTTTGAAGCTGCTTCTTCGAA-3’	30
$tRNA^{Tyr}$	5’-GGTAAAAAGAGGCCTAACCCCTGTCTTTAG-3’	30
$tRNA^{Ser(UCN)}$	5’-AAAGGAAGGAATCGAACCCCCCAAAGCTGG-3’	30
$tRNA^{Asp}$	5’-AGATATATAGGATTTAGCCTATAATTTAAC-3’	30
$tRNA^{Lys}$	5’-AAAGAGGTGTTGGTTCTCTTAATCTTTAAC-3’	30
$tRNA^{Gly}$	5’-TCTTTTTTGAATGTTGTCAAAACTAGTTAA-3’	30
$tRNA^{Arg}$	5’-TTGGTAAATATGATTATCATAATTTAATGA-3’	30
$tRNA^{His}$	5’-AAATAAGGGGTCGTAAGCCTCTGTTGTCAG-3’	30
$tRNA^{Ser(AGY)}$	5’-GAGAAAGCCATGTTGTTAGACATGGGGGCA-3’	30
$tRNA^{Leu(CUN)}$	5’-ACTTTTATTTGGAGTTGCACCAAAATTTTT-3’	30
$tRNA^{Glu}$	5’-ATTCTCGCACGGACTACAACCACGACCAAT-3’	30
$tRNA^{Thr}$	5’-TGTCCTTGGAAAAAGGTTTTCATCTCCGG-3’	30
$tRNA^{Pro}$	5’-CAGAGAAAAAGTCTTTAACTCCACCATTAG-3’	30
5S rRNA	5’-CTAATTAATTATAAGGCCAGGACCAAACCT-3’	30
